# Characterizing the shape patterns of dimorphic yeast pseudohyphae

**DOI:** 10.1098/rsos.180820

**Published:** 2018-10-17

**Authors:** Amelia Gontar, Murk J. Bottema, Benjamin J. Binder, Hayden Tronnolone

**Affiliations:** 1 Flinders Mathematical Sciences Laboratory and Medical Device Research Institute, School of Computer Science, Engineering and Mathematics, Flinders University, GPO Box 2100, Adelaide, South Australia 5001, Australia; 2 School of Mathematical Sciences, University of Adelaide, Adelaide, South Australia 5005, Australia

**Keywords:** clustered shape primitives, shape characterization, pseudohyphal growth, dimorphic yeast

## Abstract

Pseudohyphal growth of the dimorphic yeast *Saccharomyces cerevisiae* is analysed using two-dimensional top-down binary images. The colony morphology is characterized using clustered shape primitives (CSPs), which are learned automatically from the data and thus do not require a list of predefined features or *a priori* knowledge of the shape. The power of CSPs is demonstrated through the classification of pseudohyphal yeast colonies known to produce different morphologies. The classifier categorizes the yeast colonies considered with an accuracy of 0.969 and standard deviation 0.041, demonstrating that CSPs capture differences in morphology, while CSPs are found to provide greater discriminatory power than spatial indices previously used to quantify pseudohyphal growth. The analysis demonstrates that CSPs provide a promising avenue for analysing morphology in high-throughput assays.

## 1. Introduction

Yeasts are commonly known as single-celled fungi that typically grow as unconnected cells and may reproduce by budding according to a regulated pattern. On solid substrates, yeasts grow in colonies of unconnected cells; however, dimorphic yeasts, including *Saccharomyces cerevisiae* (baker’s yeast), are able to alter their growth pattern in response to external stimuli to produce chains of elongated cells. These structures, referred to as pseudohyphae, differ from true tubular hyphae observed in other fungi. The transition from the regular growth pattern to pseudohyphal growth can be triggered by low nutrient levels and represents a form of foraging [1].

**Figure 1.**
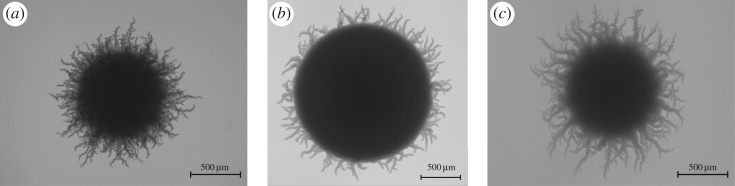
A typical image from the A7-50 (*a*), A7-500 (*b*) and AR-50 (*c*) datasets. Details of the experiments are given in Material and methods. The AWRI 796 50 µM example image was published by Binder *et al.* [7] and is licensed under a Creative Commons Attribution license (CC BY 4.0). The AWRI R2 and AWRI 796 500 µM images are reproduced courtesy of the Wine Innovation Cluster, based at the Waite Research Precinct at the University of Adelaide.

*Saccharomyces cerevisiae* is used to produce a large variety of foods, including bread, wine and beer, motivating the development of new superior strains and optimization of growth behaviour. By contrast, yeast colonies that are resistant to antimicrobial therapies may form on medical equipment, in which case it is desirable to inhibit growth [2,3]. There is thus a need to identify and classify strain-specific properties of yeast, particularly in relation to growth patterns. Yeasts are commonly studied by growing a collection of cells on a solid medium and recording a two-dimensional top-down image of the resulting colony, like those shown in figure 1, from which the morphology is analysed [4–7]. For example, high-throughput assays from genome-wide deletion mutant libraries are used to identify links between genes and changes in the growth pattern [6]. Such studies result in thousands of images, requiring robust but flexible techniques for identifying and quantifying changes in morphology.

A considerable variety of metrics have been employed to quantify the morphology of yeast colonies. Filamentous and invasive growth has been measured by the relative size of the filamentous or invasive portion of the colony, or, for invasive growth only, by comparing the pixel intensity of pre- and post-washed colonies [6,8,9]. While studies on single-gene deletion alleles have used these metrics to understand the regulation of filamentous growth [6], they provide only limited information on the pattern and growth behaviour. Both experimental and simulated data have been quantified by the relative size of the squared colony perimeter to the area, and through the coefficient of variation of the colony boundary [10]. The fractal dimension has been shown to quantify the morphologies of bacterial colonies, which possess similar colony morphologies to yeast [11,12]. The shape patterns in yeast colonies have been quantified using indices derived from normalized count functions and pair correlation functions, which have the advantage of providing information on the spatial distribution of the cells [7,13]. These studies led to successful quantification of spatial patterns in binary images of *S. cerevisiae* colonies exhibiting pseudohyphal and invasive growth; however, little work has been done to classify binary images of yeast colonies by strain or nutrient level based on the spatial patterns observed in these images.

The classification of yeast colonies into two morphological groups (smooth and fluffy) has been performed by computing 427 intensity-based and texture features and using regularized logistic regression [14]. Of the features considered, only six were identified as important for classifying the colony morphology, with the most important being the fractal dimension, average entropy texture measure within the colony, and the area of the colony structure. Such approaches are able to provide a suitably accurate classification but rely on the specification of a large number of features, most of which may not be useful or are unrelated to yeast morphology, while other important features may be overlooked. In contrast to this approach, methods have recently been developed for automatically learning the shape patterns in complex three-dimensional binary arrays [15,16]. These approaches have the advantage that important features are selected rigorously through analysis of the patterns and do not need to be specified *a priori*.

We show that yeast colony morphology may be accurately and automatically quantified using clustered shape primitives (CSPs). A shape primitive is computed at an occupied pixel in a binary image by measuring the lengths of the longest line segments, oriented at equally spaced angles, that fit entirely inside the set of occupied pixels, which represent the local shape at the pixel. Common shape patterns may be identified by applying a clustering algorithm to the set of all shape primitives computed, and the centres of the resulting clusters, referred to as CSPs, represent shape patterns that occur commonly throughout a given set of images. Importantly, CSPs are selected automatically with no need for a predetermined list of features. To demonstrate that this approach accurately captures changes in colony morphology, we use CSPs to classify two-dimensional binary images of *S. cerevisiae* colonies that are known to have different growth patterns. The datasets are chosen to allow classification by strain, nutrient concentration, and both strain and nutrient concentration simultaneously. The classification ability of CSPs is found to compare favourably to that of the indices for yeast growth developed by Binder *et al.* [7]. The classification results demonstrate that CSPs provide a new avenue for analysing morphology in high-throughput assays from genome-wide deletion mutant libraries and provide a rigorous framework for identifying changes in growth pattern.

## 2. Material and methods

### 2.1. Datasets

The experimental data used in this study were collected by Binder *et al.* [7], allowing for a direct comparison of results. The three datasets used, summarized in table 1, were chosen to illustrate the dependence of morphology on both strain and nutrient concentration. These datasets have been uploaded as part of the electronic supplementary material. Briefly, single cells of *S. cerevisiae* were used to initiate the growth of several individual yeast colonies, which were imaged successively over time. The images were converted into two-dimensional binary images using customized software, where pixels were designated as either ‘occupied’ by yeast cells or ‘unoccupied’. In order to focus on well-developed shape patterns of the colonies, we use only the last of the sequence of images for each colony.

**Table 1.** Details of the three datasets used in this study. Shown are the strains, concentrations (in micromolar), number of colonies, observation times (in hours after initiation of growth) and image resolution (in μm^2^ × pixel^−1^) of the three datasets used in the study. The name column refers to the abbreviated name of the dataset used throughout the paper.

name	strain	conc.	trials	time	res.
A7-50	AWRI 796	50	10	233	1.52
A7-500	AWRI 796	500	9	240	1.55
AR-50	AWRI R2	50	10	237	1.53

### 2.2. Clustered shape primitives

Consider data consisting of *m* groups *G*_*g*_ for *g* = 1, 2, …, *m*, such as the three experimental groups described in table 1, so that each group contains images *X* ∈ *G*_*g*_, with the full dataset denoted $G=⋃G_{g}$. For some *X* ∈ *G*_*g*_, let *Ω* be the set of pixels occupied by the yeast colony. Since the shape of the colony is of interest, only pixels on the boundary ∂*Ω* of *Ω* are analysed. For each pixel *p* ∈ ∂*Ω* and each angle *ϕ*_*d*_ = (*d* − 1)*π*/*D*, where *d* = 1,2, …, *D*, the length ${\hat{v}}_{\;p,d}$ in pixels of the longest line segment through *p* in direction *ϕ*_*d*_ lying entirely inside *Ω* is measured. This length is converted to a physical distance *v*_*p*,*d*_ so that images at different resolutions may be compared. The shape primitive associated with pixel *p* is defined to be

$$v_{p}=(v_{\;p,1},v_{\;p,2},\ldots ,v_{\;p,D}).$$

**Figure 2.**
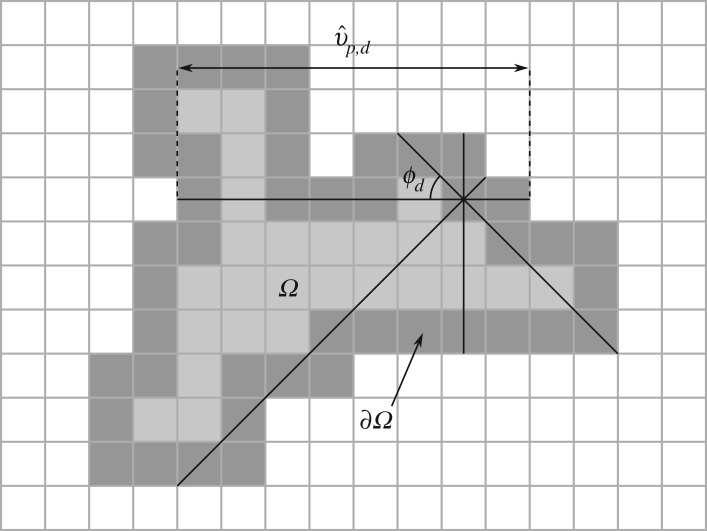
The computation of a shape primitive for *D* = 4. For a region *Ω* (all shaded squares), shape primitives are computed for each pixel *p* on the boundary ∂*Ω* (dark grey). The lengths ${\hat{\nu }}_{\;p,d}$ in pixels of the longest line segments at angles *ϕ*_*d*_ = (*d* − 1)*π*/*D*, *d* = 1,2, …, *D*, to the horizontal axis lying entirely inside *Ω* and passing through *p* are measured, where *D* = 4. The associated shape primitive comprises these lengths converted to physical units, denoted $\nu_{p}=(\nu_{\;p,1},\nu_{\;p,2},\ldots ,\nu_{\;p,D})$.

The computation of a shape primitive for one *p* ∈ ∂*Ω* with *D* = 4 is illustrated in figure 2.

**Figure 3.**
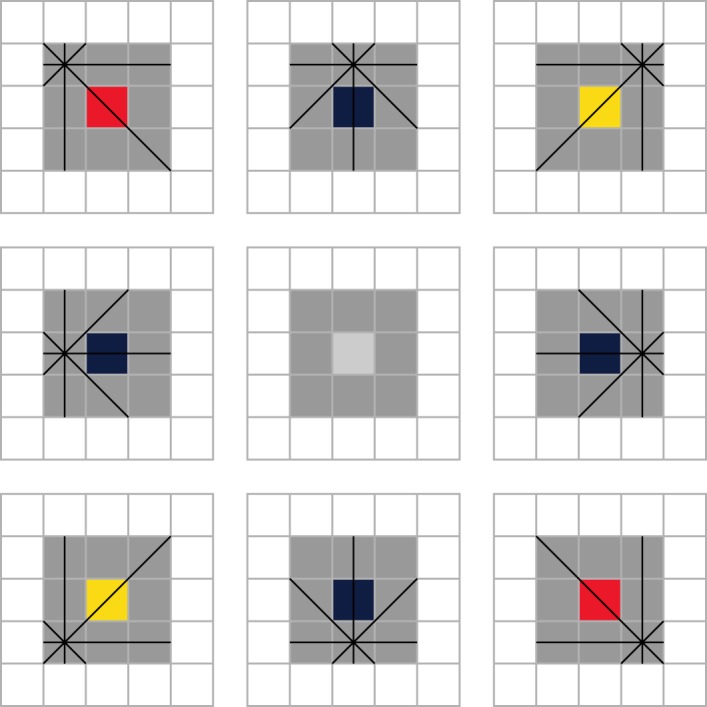
The shape primitives for a square with sides of length three pixels (shaded cells), shown in the centre of the image with the border highlighted (darker pixels). This shape yields three unique shape primitives: $\left(3,\sqrt{2},3,3\sqrt{2}\right)$ (red), $\left(3,2\sqrt{2},3,2\sqrt{2}\right)$ (blue) and $\left(3,3\sqrt{2},3,\sqrt{2}\right)$ (yellow), illustrated in the corresponding diagrams.

To demonstrate how shape primitives represent the local shape of an object, we consider the computation of all shape primitives for a square with sides of length three pixels, shown in figure 3. The boundary of this shape comprises eight pixels, so that only the central pixel is not included, and a shape primitive with *D* = 4 is computed for each of these. This produces the three unique shape primitives $\left(3,\sqrt{2},3,3\sqrt{2}\right)$, $\left(3,2\sqrt{2},3,2\sqrt{2}\right)$ and $\left(3,3\sqrt{2},3,\sqrt{2}\right)$, which together describe the local shape of the square and form the basis for the classification method used here.

The representation space *F*_*g*_ for a group *G*_*g*_ is the collection of all shape primitives computed from objects in *G*_*g*_; that is,

$$F_{g}=\{v_{p}:\;p\in X,X\in G_{g}\}.$$

The Euclidean distance between two vectors is used to measure similarity, and similar elements in *F*_*g*_ represent common local shape patterns appearing in *G*_*g*_, which are identified using *k*-means clustering. The cluster centres are referred to as CSPs. The CSPs for data group *G*_*g*_ are vectors $C_{k}^{g}$ for *k* = 1, 2, …, *K*, which have the same dimension as the shape primitives $v_{p}$. The *m* groups each have *K* CSPs, resulting in *M* = *Km* CSPs in total. For convenience, these are labelled sequentially *C*_*i*_ for *i* = 1,2, …, *M*, where $C_{(g-1)K+k}=C_{k}^{g}$ for *g* = 1, 2, …, *m* and *k* = 1, 2, …, *K*, while the entire collection of *M* CSPs is denoted C.

The distribution of line segments lengths within the object *Ω* at various orientations *ϕ*_*d*_, *d* = 1, 2, …, *D*, allows discrimination between shapes. In the context of yeast colonies, if all the pseudohyphae consist of straight radial spikes of the same length, then the distribution of line segment lengths would be a uniform function of *ϕ*_*d*_. The distribution of line segment lengths at various orientations, regardless of the frame of reference, provides a measure of the deviation from this model. In other words, it is not the orientation of a single line segment that matters, it is the distribution of these that defines shape patterns.

The shape of each object may be summarized by grouping all of its associated shape primitives. For each *p* ∈ ∂*Ω* for an image *X*, let

$$Y\text{(}p\text{)}=\;\underset{i\in \{1\text{,}\;2\text{,}\;\ldots \text{,}\;M\}}{\text{arg}\;\;\text{min}}\{∥C_{i}-v_{p}∥\},$$

where ‖ · ‖ is the Euclidean norm. The object *Y* is the CSP map associated with *X*. The normalized histogram of CSP labels associated with *X* is

2.1 $$h_{X}=(h_{1},h_{2},\ldots ,h_{M}),$$

where *h*_*i*_ is the proportion of labels in *Y* with value *i*. The histogram $h_{X}$ represents the shape content of the image *X* and provides a quantitative description of the shape characteristics of the image. CSPs may be thought of as a descendant of the bag-of-words model, where each CSP is a visual word [17,18].

While it would be possible to instead analyse the representation space using principal component analysis (PCA), this is not a useful choice in the context of this work. While PCA is useful for finding the direction (eigenvector) of greatest information content, this is not necessarily the direction of optimal classification. Furthermore, *k*-means clustering, as used in this method, does not reduce the number of dimensions but rather transforms the data into CSP features, so cannot be replaced directly by another method for dimension reduction.

### 2.3. Spatial indices

The classification power of CSP-based features was compared with that of three spatial indices previously used to quantify yeast colonies [7], which are described here briefly.

A normalized count function is used to identify the radius *R*_CSR_ at which the probability of finding a pixel is equal to the value for pixels distributed with complete spatial randomness (CSR), while the maximum radius of the colony is denoted *R*. The level of filamentation in the radial direction is measured by the radial index *I*_*r*_ = 1 − *R*_CSR_/*R* ∈ [0, 1]. The angular variation is measured by computing the distribution of angles to each pixel measured from the *x*-axis and relative to the colony centroid, normalized by the corresponding values at the CSR state, and computing the variance *I*_*θ*_. This is referred to as the angular index and is related to boundary fluctuation, which has been used to describe yeast biofilms [10]. Local aggregation is measured by computing the angular pair-correlation function *F*_*Θ*_ for the pixels and introducing *I*_*Θ*_ = *F*_*Θ*_(1) − 1, which is referred to as the pair-correlation index.

Here, the number of bins used for the radial, angular and pair-correlation indices were 178, 200 and 200, respectively, to match the values used in the original study [7]. Since the number of calculations needed for the pair-correlation index is computationally expensive, and to ensure consistency with the original study, this index was computed using 1000 pixels randomly sampled from the colony.

### 2.4. Classification using clustered shape primitives and spatial indices

To demonstrate that CSPs identify differences in morphology, three classification problems were performed on the test data: two different strains with the same nutrient concentration (using A7-50 and AR-50); the same strain grown at different nutrient concentrations (using A7-50 and A7-500); and any of the three types of yeast colony (a three-group classification problem comprising A7-50, A7-500 and AR-50). Both CSP-based features and spatial indices were used to classify yeast colonies so as to compare performance. Within each problem, the images from each dataset were split evenly between the training and testing groups at random, except for A7-500, for which five images were used for training with four left for the testing set. All data and code used for this analysis has been made available [19].

The information from the CSPs and spatial indices was combined into the augmented feature vector

$$h_{j}^{⋆}=\left(h_{\;j,1},h_{\;j,2},\ldots ,h_{\;j,M},\frac{I_{r}}{M},\frac{I_{\theta }}{M},\frac{I_{\Theta }}{M}\right),$$

where the *h*_*j*_ are as in the original normalized histogram (2.1) and *M* = *Km* is the number of CSPs. The factor 1/*M* ensures that all features are of the same magnitude. Using these augmented feature vectors as input, linear discriminant analysis [20] was used for classification. Only one feature was used for classification in order to avoid overfitting, since the number of images in the testing sets was small.

Within each classification problem, three methods of testing and analysing the classifier were considered. First, the images from each group were split as evenly as possible between training and testing sets. The classifier was trained and tested once for each individual feature, and the best feature and the corresponding highest classification score were recorded. The *k*-means clustering step was repeated *n* = 30 times. The mean and standard deviation of the classification accuracies were recorded, along with the number of times each of the spatial indices were chosen as a best feature. Since the CSP labels and values were different for each repetition, it was not possible to record the number of times these were chosen as a best feature.

Second, leave-one-out cross-validation (LOOCV) was conducted [20,21]. Let *S* denote the number of images in a dataset. A single image was removed from the training data and set aside as the test image. The remaining *S* − 1 images were used to determine the single best feature for classification by exhaustively testing each feature separately. The resulting classifier was used to classify the test image. This classification was either correct or incorrect. By repeating this process, including the feature selection step, *S* times with each image taking a turn as the test image, a classification result was obtained for each image. The accuracy was taken to be the proportion of images classified correctly. The number of times each spatial index was chosen as the best feature was also recorded.

The above two methods for testing the classifier give classification scores, but do not necessarily shed any light on which shape patterns differentiate well between colonies. To address this, in the feature analysis method the training and testing method was repeated only once (*n* = 1), and the features that gave a high classification test score were recorded. If the best features are the occurrences of a CSP, then this means certain CSPs occur more frequently in one group of images than another. A given CSP may be visualized using a histogram of the feature vector. Although this method of evaluating the classifier is not as robust as training and testing or LOOCV, it allows for characterization of the important shape patterns that discriminate well between yeast colonies.

For each of the three classification problems, the training and testing, and LOOCV methods were repeated with the number of angles used to compute the CSPs set at *D* = 4 and 12, and with *K* = 5 and 10 clusters per class. The feature analysis method was then applied to the best combination of *D* and *K* from the training and testing method to determine the best features.

A large number of measures, in addition to the spatial indices considered here, are available for quantifying colony shape [10,14]. It has been found that these are generally highly correlated with the spatial indices and may not provide additional classification power. For example, the perimeter to area measure P2A [10] is qualitatively similar in behaviour to the spatial indices, and it has been found that adding P2A to the augmented feature vector, along with the spatial indices, does not provide a significant improvement in the classification. For this work, we thus include only the spatial indices, which are representative of standard measures computed without using CSPs. Results including both the spatial indices and P2A are available in the electronic supplementary material.

## 3. Results

### 3.1. Classification by strain

The results of classifying the yeast datasets A7-50 and AR-50 according to strain are summarized in table 2. The best accuracy score was achieved using *D* = 4 and *K* = 10; however, this classification accuracy was not significantly different to those obtained using *D* = 4, *K* = 5 or *D* = 12, *K* = 10. The results suggest that measuring the lengths of the yeast colonies at only four angles provides sufficient information for classification using features based on CSPs. Choosing a higher number of clusters per class appears to be more important than using a higher number of angles at which to make length measurements; that is, increasing the number of components in each shape primitive appears to be less important than increasing the number of clusters.

**Table 2.** The mean and standard deviation (*n* = 30) as a function of the number of clusters *K* per class and number of angles *D* chosen to compute the CSPs when classifying A7-50 and AR-50 by strain. The best classification accuracy was achieved with *D* = 4 and *K* = 10.

*K*	*D* = 4	*D* = 12
5	*μ* = 0.987, *σ* = 0.035	*μ* = 0.907, *σ* = 0.037
10	*μ* = 0.997, *σ* = 0.018	*μ* = 0.987, *σ* = 0.035

During training and testing, the spatial indices were never chosen as one of the best features for classification. At approximately 240 h after initiation of growth, the values of the indices did not discriminate well between A7-50 and AR-50, which is consistent with previous results [7].

During LOOCV, all 20 images were classified correctly regardless of the number of angles *D* at which the CSPs were computed and the number of clusters *K* per class, as can been seen from the results in table 3. This means that, for all 20 images, there was at least one feature (either based on a CSP or spatial index) that could classify that image correctly.

**Table 3.** The accuracy score as a function of the number of clusters *K* per class and number of angles *D* used to compute the CSPs when performing LOOCV for the classification of A7-50 and AR-50 by strain.

*K*	*D* = 4	*D* = 12
5	1	1
10	1	1

Under LOOCV, the angular index and pair-correlation index were picked as one of the best features six and eight times, respectively, as per table 4. This does not necessarily mean that these spatial indices were the only features that correctly classified these images, since there may have been more than one feature that gave a correct classification. The number of times each index achieved a perfect classification accuracy is independent of the choices for *D* and *K*, as these affect the computed CSPs only. These results demonstrate that a combination of shape features based on CSPs and spatial indices can classify yeast colonies by strain to a very high degree of accuracy.

**Table 4.** The number of times out of the 20 repetitions that each of the spatial indices was chosen as one of the best features when classifying A7-50 and AR-50 by strain and performing LOOCV.

index	*I*_*r*_	*I*_*θ*_	*I*_*Θ*_
times chosen	0	6	8

**Figure 4.**
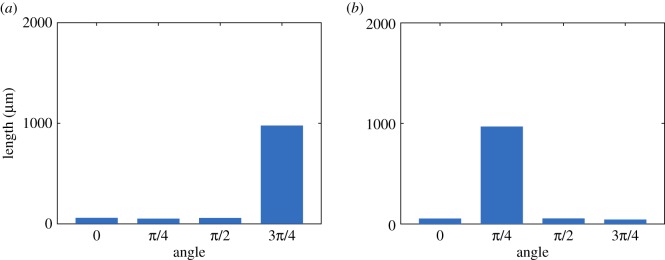
An illustration of the shape patterns that gave a perfect classification of A7-50 and AR-50 by strain with *D* = 4 and *K* = 10. Each bin represents an angle, and the height of the bin represents the length measurement made at that angle (after clustering). Shown are CSPs 6 (*a*) and 8 (*b*).

Although not a robust test of the classifier, the feature analysis method allows for visualization of the shape patterns that result in good discrimination between the groups. In order to analyse the features that gave the best discrimination between strains, the training and testing method was repeated once with *D* = 4 and *K* = 10, since these gave the best classification accuracy during training and testing with *n* = 30 (table 2). Out of the 23 features tested, two gave a perfect classification score and are shown in figure 4. These CSPs are (59.7, 52.1, 59.3, 977.6) and (54.1, 968.9, 55.0, 44.3), where each entry in the CSP vectors represents a length in micrometres, measured at angles of 0, *π*/4, *π*/2 and 3*π*/4, respectively. The largest length measurements in these CSPs were approximately equal in size and occurred at the angles 3*π*/4 and *π*/4, respectively, with the two represented at comparable levels. The largest length measurement is approximately 970 µm for both shape features. These features are picking out longer filaments present in the yeast colonies at these angles.

### 3.2. Classification by nutrient concentration

The yeast colonies from A7-50 and A7-500 were classified by nutrient concentration by splitting the data into training and testing sets and conducting *n* = 30 trials, with the results summarized in table 5. All combinations of values for *D* and *K* gave a perfect classification, while the spatial indices were never chosen as a best feature. Examining the original images reveals that the A7-500 group features a more uniform growth pattern than the A7-50 group, as evident in the examples from figure 1.

**Table 5.** The mean and standard deviation (*n* = 30) as a function of the number of clusters *K* per class and number of angles *D* used to compute the CSPs when classifying A7-50 and A7-500 by nutrient concentration.

*K*	*D* = 4	*D* = 12
5	*μ* = 1.00, *σ* = 0.00	*μ* = 1.00, *σ* = 0.00
10	*μ* = 1.00, *σ* = 0.00	*μ* = 1.00, *σ* = 0.00

Similarly, during LOOCV, all choices for *D* and *K* gave a perfect classification, as per the results given in table 6. For each of the 19 images, there was at least one individual feature that classified that image correctly.

**Table 6.** The accuracy score obtained using LOOCV when classifying A7-50 and A7-500 by nutrient concentration, as a function of the number of angles *D* used to compute the CSPs and the number of clusters *K* per class.

*K*	*D* = 4	*D* = 12
5	1	1
10	1	1

The number of times each spatial index was selected is given in table 7, which shows that the radial index is selected more than twice as often as either of the other indices. This is not surprising, as the growth patterns in the A7-500 group appear to be more circular in shape with shorter pseudohyphae than those in the A7-50 group, so the CSR radius *R*_CSR_ is expected to be higher for the colonies in the A7-500 group. In general, radial information appears to be more important than angular information when using the spatial indices to discriminate between yeast colonies based on nutrient concentration.

**Table 7.** The number of times (out of 19 runs) each of the spatial indices was chosen as one of the best features for classification when performing LOOCV for the classification of A7-50 and A7-500 by nutrient concentration.

index	*I*_*r*_	*I*_*θ*_	*I*_*Θ*_
times chosen	15	6	5

**Figure 5.**
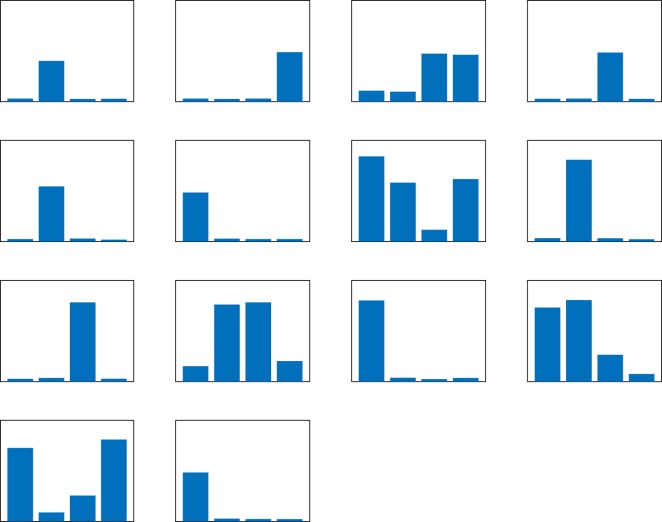
An illustration of all of the shape patterns that gave a perfect classification of A7-50 and A7-500 with *D* = 4 and *K* = 10. In each histogram, the bins represent the angles 0, *π*/4, *π*/2, 3*π*/4 (left to right), and the height of each bin represents the length measurement made at that angle (after clustering). In each panel, the range of the vertical axis is 0–2000 µm.

Since all combinations of values of *D* and *K* performed equally well during training and testing, *D* = 4 and *K* = 10 were chosen for feature analysis for consistency with the classification by strain. Of the 23 features tested, 14 of the CSPs gave a perfect classification accuracy, and are illustrated in figure 5. None of the spatial indices achieved a similar classification accuracy. As before, the largest length measurements of several of the best CSP-based features often occurred at the angles *π*/4 and 3*π*/4.

### 3.3. Classification by strain and nutrient concentration

The yeast colonies were classified by both strain and nutrient concentration using all three datasets by splitting the data into training and testing sets and conducting *n* = 30 trials, with the results summarized in table 8. The best classification score was achieved with *D* = 12 and *K* = 10; however, this score was not significantly different from those obtained using (*D*, *K*) = (4, 5) or (*D*, *K*) = (4, 10). Increasing both the number of angles *D* (the number of elements in the shape feature vectors) and the number of clusters *K* per class simultaneously produces a slight increase in the effectiveness of the classifier. The spatial indices were never chosen as a best feature, which suggests that shape-based features are more important when classifying yeast colonies by both strain and nutrient concentration.

**Table 8.** The mean *μ* and standard deviation σ of the classification accuracy with *n* = 30 trials as a function of the number of clusters *K* per class and number of angles *D* used to compute the CSPs for the three-group classification problem using A7-50, A7-500, AR-50. The best classification accuracy was achieved using *D* = 12 and *K* = 10.

*K*	*D* = 4	*D* = 12
5	*μ* = 0.957 , *σ* = 0.052	*μ* = 0.898, *σ* = 0.064
10	*μ* = 0.955, *σ* = 0.064	*μ* = 0.969, *σ* = 0.041

During LOOCV, all 29 images were classified correctly regardless of the choices of *D* and *K*, as per the results in table 9. This means there was at least one feature that could classify each image correctly, suggesting the classifier built here using a combination of features based on CSPs and spatial indices extracts useful information from the binary yeast colonies.

**Table 9.** The accuracy score obtained using LOOCV for the three-group classification problem as a function of the number of clusters *K* per class and number of angles *D* used to compute the CSPs using A7-50, A7-500 and AR-50.

*K*	*D* = 4	*D* = 12
5	1	1
10	1	1

The number of times that each spatial index was chosen as a best feature is shown in table 10. These results are not necessarily achieved exclusively by that feature, as other (CSP-based) features may have resulted in an equal best classification. The number of times each spatial index gave a perfect accuracy score was independent of the choices for *D* and *K*.

**Table 10.** The number of times (out of 29 runs) each of the spatial indices was chosen as one of the best features for classification, using LOOCV for the three-group classification problem using A7-50, A7-500 and AR-50.

index	*I*_*r*_	*I*_*θ*_	*I*_*Θ*_
times chosen	6	1	2

**Figure 6.**
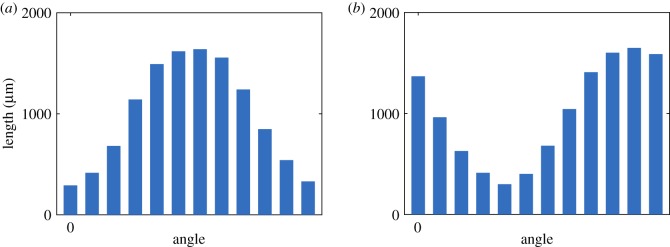
An illustration of the CSPs that gave a perfect classification when classifying A7-50, A7-500 and AR-50 by both strain and nutrient concentration using *D* = 12, corresponding to bin widths *π*/12. The height of each bin represents the length measurement made at the corresponding angle. Shown are CSPs 11 (*a*) and 19 (*b*).

In order to analyse the features, *D* = 12 and *K* = 10 were chosen for one run of the classifier, since this combination achieved the best accuracy during training and testing. Of the 33 features tested, two CSPs achieved a perfect classification accuracy and are shown in figure 6. None of the spatial indices produced a perfect accuracy score.

The shapes corresponding to the CSP-based features cannot be compared directly to those from the other classification problems shown in figures 4 and 5 because different values of *D* and *K* were used to compute the features, which means that the features vectors are of different size. Despite this, similar patterns can be seen. For instance, the CSP labelled 19 features a similar distribution of length as a function of angle to that of CSP labelled 6 in figure 4, with longer lengths around an angle of 3*π*/4 and shorter lengths around *π*/4. Overall, for the classification of yeast colonies based on strain and nutrient concentration, a binary shape pattern with the largest length measurement oriented at an angle of 3*π*/4 appears to give the most discrimination between classes, which probably occurs because these measurements are picking out differences in filament lengths between the groups.

## 4. Discussion

A combination of CSP-based features and spatial indices have been used to classify two-dimensional top-down binary images of filamentous yeast colonies. The colonies were classified by strain, nutrient concentration, and both strain and nutrient concentration simultaneously. The CSPs were based on the shape of the colony instead of the texture, and the important CSP-based features were learned automatically from the data. A high classification accuracy was achieved in each of the three problems considered, demonstrating that CSPs represent a promising tool for quantifying colony morphology. The classifier also provides a means of identifying and quantifying the specific shape patterns that lead to a strong classification, which provides a greater understanding of the binary shape patterns occurring in yeasts colonies.

Whereas previous classification methods have made use of texture features derived from the interior of the colony [14], the classifier presented here uses only binary information from the boundary. The average classification accuracy for the two-group classification problem where the yeast colonies from A7-50 and AR-50 were classified by strain was comparable to the two-group morphological classification problem of smooth and fluffy colonies by Ruusuvuori *et al.* [14] (0.997 compared with 0.988), indicating that the method presented here is equally as powerful. Past analyses have also required extensive lists of features to be identified as input to the classification in order to identify which were useful. Despite the size of such lists, this approach may result in important features being overlooked and thus not considered by the classifier. The method presented here has the advantage of selecting the best features automatically, avoiding the need to specify features, which risks missing key attributes.

The shape-only approach presented here is appropriate when full grey-scale information is not available but does not supersede existing methods when different data is available. Although the method was tuned to the specific attributes of the data motivating this study, there are many situations in which shape-only data is the natural setting. This is the case when analysing three-dimensional objects that are locally so irregular in shape that standard geometric descriptions are not practical. An example is the trabecular structure of cancellous bone [15].

The automatic selection of features means that the method developed here has great potential to assist in the analysis of high-throughput assays from genome-wide deletion mutant libraries by providing a rigorous framework for identifying differences in the morphologies of different mutants. For example, CSPs could be used to group mutants based on changes to morphology, which would reveal links between particular genes and colony shape. Furthermore, the automatic feature selection means that the techniques developed here could be used to analyse a variety of other microbial-colony images, such as other fungi or bacteria.

## Supplementary Material

Results Including P2A

## Data accessibility

The datasets and code used for this study are available online from Figshare Digital Repository (doi:10.4225/55/5b0605866669e) under a Creative Commons Attribution licence (CC BY 4.0).
