# Supplementary material for: Characterizing the shape patterns of dimorphic yeast pseudohyphae
Source: R Soc Open Sci. 2018 Oct 17;5(10):180820. doi: 10.1098/rsos.180820 (PMC6227998; doi:10.1098/rsos.180820)
Supplement: Results Including P2A [file rsos180820supp1.pdf]

# Characterising the shape patterns of dimorphic yeast pseudohyphae (Supplementary Material)

**Amelia Gontar[1], Murk J. Bottema[1], Benjamin J. Binder[2], and Hayden Tronnolone*[2]**

[1]Flinders Mathematical Sciences Laboratory and Medical Device Research Institute, School of Computer Science, Engineering and Mathematics, Flinders University, GPO Box 2100, Adelaide SA 5001, Australia
[2]School of Mathematical Sciences, University of Adelaide, Adelaide, SA 5005, Australia

October 5, 2018

# 1 Results Including P2A

## 1.1 Formulation

Yeast colonies have been quantified using P2A (Chen, Noorbakhsh, Adams, Samaniego-Evans, Agollah, Nevozhay, Kuzdzal-Fick, Mehta and Balázsi, 2014), which compares the perimeter $p$ with the inclosed area $a$ and is the inverse of the isoperimetric quotient. This quantity is defined

$$P = \frac{p^2}{4\pi a}.$$

It is known that $P \geq 1$ with equality if and only if the shape considered is a circle. To examine the classification power of P2A, all training, testing, and LOOCV experiments were repeated with this quantity included. The augmented feature vector for image $j$ was

$$\boldsymbol{h}_j^\star = \left( h_{j,1}, h_{j,2}, \ldots, h_{j,M}, \frac{P}{MP_M}, \frac{I_r}{M}, \frac{I_\theta}{M}, \frac{I_\Theta}{M} \right),$$

where $P_M$ is the maximum value of P2A across all yeast colony images considered in the study. Dividing by $P_M$ ensures that $0 \leq P \leq 1$, and dividing by $M$ ensures that all features are of the same magnitude. There was no need to repeat the feature analysis method because this involved CSPs only. Table numbers in this section match those in the main manuscript.

*Corresponding author: hayden.tronnolone@adelaide.edu.au

## 1.2 Classification by strain

Table 2: The mean and standard deviation ($n = 30$) as a function of the number of clusters $K$ per class and number of angles $D$ chosen to compute the CSPs when classifying A7-50 and AR-50 by strain. The best classification accuracy was achieved with $D = 4$ and $K = 10$.

| $K$ | 4 angles | 12 angles |
|---|---|---|
| 5 | $\mu = 0.990,\ \sigma = 0.031$ | $\mu = 0.910,\ \sigma = 0.031$ |
| 10 | $\mu = 0.997,\ \sigma = 0.018$ | $\mu = 0.983,\ \sigma = 0.038$ |

For training and testing: $D = 4, K = 5$, none of the indices chosen. $D = 4, K = 10$, none of the indices chosen. $D = 12, K = 5$, none of the indices chosen. $D = 12, K = 10$, none of the indices chosen.

Table 3: The accuracy score as a function of the number of clusters $K$ per class and number of angles $D$ used to compute the CSPs when performing LOOCV for the classification of A7-50 and AR-50 by strain.

| $K$ | 4 angles | 12 angles |
|---|---|---|
| 5 | 1.00 | 1.00 |
| 10 | 1.00 | 1.00 |

Table 4: The number of times (out of 20 runs) each of the spatial indices was chosen as one of the best features when classifying A7-50 and AR-50 by strain and performing LOOCV.

| Index | P2A | $I_r$ | $I_\theta$ | $I_\Theta$ |
|---|---|---|---|---|
| Times chosen | 10 | 0 | 6 | 8 |

## 1.3 Classification by nutrient concentration

For training and testing: $D = 4, K = 5$, none of the indices chosen. $D = 4, K = 10$, none of the indices chosen. $D = 12, K = 5$, none of the indices chosen. $D = 12, K = 10$, none of the indices chosen.

Table 5: The mean and standard deviation ($n = 30$) as a function of the number of clusters $K$ per class and number of angles $D$ used to compute the CSPs when classifying A7-50 and A7-500 by nutrient concentration.

| $K$ | 4 angles | 12 angles |
|---|---|---|
| 5 | $\mu = 1.00,\ \sigma = 0.00$ | $\mu = 1.00,\ \sigma = 0.00$ |
| 10 | $\mu = 1.00,\ \sigma = 0.00$ | $\mu = 1.00,\ \sigma = 0.00$ |

Table 6: The accuracy score obtained using LOOCV when classifying A7-50 and A7-500 by nutrient concentration, as a function of the number of clusters $K$ per class and number of angles $D$ used to compute the CSPs.

| $K$ | 4 angles | 12 angles |
|---|---|---|
| 5 | 1.00 | 1.00 |
| 10 | 1.00 | 1.00 |

Table 7: The number of times (out of 19 runs) each of the spatial indices was chosen as one of the best features for classification when performing LOOCV for the classification of A7-50 and A7-500 by nutrient concentration.

| Index | P2A | $I_r$ | $I_\theta$ | $I_\Theta$ |
|---|---|---|---|---|
| Times chosen | 17 | 15 | 6 | 5 |

## 1.4 Classification by strain and nutrient concentration

Table 8: The mean and standard deviation ($n = 30$) of the classification accuracy as a function of the number of clusters $K$ per class and number of angles $D$ used to compute the CSPs for the three-group classification problem using A7-50, A7-500, AR-50. The best classification accuracy was achieved using $D = 12$ and $K = 10$.

| $K$ | 4 angles | 12 angles |
|---|---|---|
| 5 | $\mu = 0.955,\ \sigma = 0.058$ | $\mu = 0.912,\ \sigma = 0.067$ |
| 10 | $\mu = 0.917,\ \sigma = 0.073$ | $\mu = 0.971,\ \sigma = 0.036$ |

For training and testing: $D = 4, K = 5$, none of the indices chosen. $D = 4, K = 10$, none of the indices chosen. $D = 12, K = 5$, none of the indices chosen. $D = 12, K = 10$, none of the indices chosen.

Table 9: The accuracy score obtained using LOOCV for the three-group classification problem as a function of the number of clusters $K$ per class and number of angles $D$ used to compute the CSPs using A7-50, A7-500, and AR-50.

| $K$ | 4 angles | 12 angles |
|---|---|---|
| 5 | 1.00 | 1.00 |
| 10 | 1.00 | 1.00 |

Table 10: The number of times (out of 29 runs) each of the spatial indices was chosen as one of the best features for classification, using LOOCV for the three-group classification problem using A7-50, A7-500 and AR-50.

| Index | P2A | $I_r$ | $I_\theta$ | $I_\Theta$ |
|---|---|---|---|---|
| Times chosen | 15 | 6 | 1 | 2 |

## 1.5 Summary

For the training and testing experiments, the feature $P/MP_M$ was never chosen as a best feature. The values of $D$ and $K$ that result in the best classification result have not changed. For LOOCV, the feature $P/MP_M$ was chosen as a best feature more often than either the radial, angular, or angular pair-correlation indices. Although P2A appears to have some discriminatory power, including this feature did not improve the classification results during LOOCV because, for each image that was left out, there was already a feature that had classified it correctly.

Overall, incorporating P2A as a feature did not improve the classification. This can be explained, in part, by comparing P2A with the indices used in the paper, as shown in figure 1. While P2A and the indices each measure different quantities, these all display the same qualitative behaviour and are highly correlated.

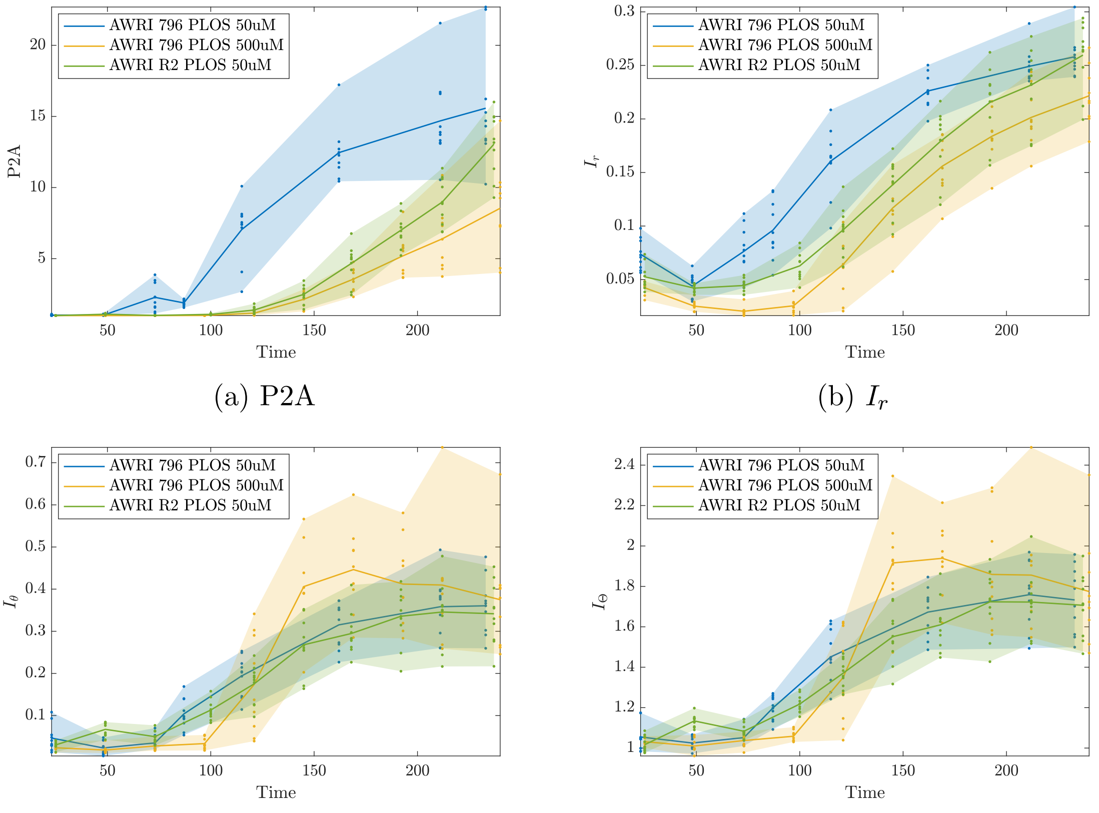


(a) P2A

(b) $I_r$

(c) $I_\theta$

(d) $I_\Theta$

Figure 1: P2A, $I_r$, $I_\theta$ and $I_\Theta$ computed for the images used in this study. Each image is represented by a coloured dot, the mean over time is shown as a solid line, and the range for each strain in shaded in the same colour. The ranges of the indices $I_r$, $I_\theta$ and $I_\Theta$ overlap. A similar overlap is seen for P2A and P2A is highly correlated with the spatial indices.

# References

Chen, L., Noorbakhsh, J., Adams, R. M., Samaniego-Evans, J., Agollah, G., Nevozhay, D., Kuzdzal-Fick, J., Mehta, P. and Balázsi, G. (2014), 'Two-dimensionality of yeast colony expansion accompanied by pattern formation', *PLOS Computation Biology* **10**(12), e1003979.
